# Study on Laser Parameter Measurement System Based on Cone-Arranged Fibers and CCD Camera

**DOI:** 10.3390/s22207892

**Published:** 2022-10-17

**Authors:** Jie Luo, Laian Qin, Zaihong Hou, Silong Zhang, Wenyue Zhu, Wenlu Guan

**Affiliations:** 1 School of Environmental Science and Optoelectronic Technology, University of Science and Technology of China, Hefei 230026, China; 2 Key Laboratory of Atmospheric Optics, Anhui Institute of Optics and Fine Mechanics, Hefei Institutes of Physical Science, Chinese Academy of Sciences, Hefei 230031, China; 3 State Key Laboratory of Pulsed Power Laser Technology, Hefei 230037, China

**Keywords:** laser parameter, array fibers, CCD camera, integrated optics, PIB

## Abstract

This paper proposes a new laser parameter measuring method based on cone-arranged fibers to further improve the measurable spot size, allowable incident angle range, and spatial sampling resolution. This method takes a conical array composed of flexible fibers to sample and shrink the cross-section spot of the laser beam, facilitating low-distortion shooting with a charge-coupled diode (CCD) camera, and adopts homogenized processing and algorithm analysis to correct the spot. This method is experimentally proven to achieve high-accuracy measurements with a decimeter-level spot-receiving surface, millimeter-level resolution, and high tolerance in order to incite skew angle. Comparing the measured spot under normal incidence with the real one, the root mean square error (RMSE) of their power in the bucket (PIB) curves is less than 1%. When the incident angle change is between −8° and 8°, the RMSE is less than 2% and the measurement error of total power is less than 5% based on the premise that the fiber’s numerical aperture (NA) is 0.22. The possibility of further optimizing the measurement method by changing the fiber parameters and array design is also reported.

## 1. Introduction

The laser parameter is an essential indicator in the scientific evaluation of laser systems. Typical laser parameters, such as beam width, β factor, and M^2^ factor, can be obtained by measuring the intensity distribution of one or more axial cross-section spots [1,2,3]. When the spot is large, the measurement system prioritizes the array target composed of attenuation and detection units [4,5,6,7]. The system always has a large aperture, and the calibration of the whole target can conveniently correct the inconsistent responsivity of sampling units. However, its resolution is often limited by the complex unit design. Moreover, the transmittance and response of the units are sensitive to the incident angle in many applications [5,6,7], which is regarded as a significant error source. Since there are frequently variable and unmeasurable skew angles between the beam and the receiving system caused by alignment error, the measurement is expected to be only related to the incident energy, not the incident angle. When the spot is small, a CCD camera is commonly used [8,9,10,11,12,13,14]. The beam can act directly on the CCD array in the camera [8,9] or be imaged on various screens and shot by the camera [1,10,11]. Such methods have the advantage of high resolution [12,13]. However, on the one hand, shooting requires precise space alignment, so fast servo equipment is necessary [12,14], which adds difficulty to practical applications. On the other hand, the measurable spot size is limited by distortion, which is easily caused by the large field of view. Considering these factors, if the cross-section spot is shrunk with high fidelity before shooting, the large spot is measurable by the CCD camera without severe distortion. For the shrinking system, the input maintains a large aperture to facilitate the alignment of the beam, and the reduced output can be relatively fixed to the camera. Compared with the traditional CCD camera shooting, this method combines part of the structure of the array target, such as the large target surface and the sampling units, which not only increases the measurable spot size but also reduces the difficulty of beam alignment and improves the freedom of integration. Compared with the measurement of array targets, this method uses a single attenuator and a commercial CCD camera instead of multiple attenuators and detector units, simplifying the design. In addition, the main factor limiting the resolution changes from the outer diameter of the attenuator or detector to the sampling unit, leading to a significant increase in resolution. In summary, this method of combining the two approaches is of significance to optimize the measurement system.

To achieve the above purpose, this paper proposes taking a cone-arranged fiber array to sample and shrink the beam. The beam propagation path is converted from free space to the optical waveguide. Accordingly, the high-fidelity shrinking of the spot can be achieved through the high-uniform transmission of the fiber array. The fiber can have both sampling and transmission functions. As the sampling unit, it can control the spatial resolution to the level of the microscopic fiber outer diameter [15,16] and increase the system’s allowable incident angle because of the total reflection transmission in the core [17,18]. As the transmission unit, the fiber has a high macro bending transmittance [19] and laser-induced damage threshold [20,21], which help the system adapt to the measurement and evaluation of multiple types of lasers. To prove the feasibility of this application, this paper invents a laser parameter measurement system based on it. The protection target plate with sparsely arranged fibers is used as the input, and the tightly arranged fiber array at the other end is used as the output. Unlike the traditional array target, in order to pursue the robustness of measurement when the incident angle changes and the simplification of the system, there is no diaphragm array behind the fibers but a single filter and attenuator. In this case, the array beams output by fibers cannot avoid mutual and complex crosstalk; thus, a homogenizer is set to unify the crosstalk. After the CCD camera shoots the spot imaged on the homogenizer, the intensity distribution can be computationally corrected by average pooling, crosstalk removal, and interpolated beam expansion. Simultaneously, the total power and the power density distribution can be obtained through pre-calibration, and the required laser parameters can be further calculated.

To verify the accuracy of the measurement, the experiment compares the cross-section spot measured by our system with the spot imaged on the diffuse reflector, which can be seen as the real one. The results show that the RMSE of the PIB curves between the two spots, which characterizes the intensity distribution, is less than 1%. An adjustable light source and a power meter are used to calibrate the system: a coefficient of determination of the linear fit of 0.99979 is obtained, verifying the reliability of the total power measurement. Furthermore, measurements at different incident angles are also carried out. Under the premise that the theoretical NA of the fiber is 0.22, the change in the measured total power is less than 2% in the interval of −6°~6°, and the RMSE of PIB is less than 1%; in the interval of –8°~8°, these values are less than 5% and 2%, respectively. These results quantify the allowable incident angle range under different measurement requirements. Finally, we propose the possibility that fiber with a greater NA can further increase the allowable incident angle range.

## 2. Theoretical Model

### 2.1. Bend Fiber Transmission

In this application, the fiber is expected to meet the following conditions:

1. All-glass fiber is suitable for its high laser damage threshold due to low absorption and high-temperature resistance.
2. Multimode fiber is more suitable than single-mode fiber because of its larger core diameter and higher laser damage tolerance threshold. The former has a higher duty cycle after the array is integrated, so the spot details the are more accessible [22].
3. The bending diameter of the fiber should be at least in the order of centimeters to minimize the macro bending loss [23,24].
4. The ring-shaped cladding face should not be exposed to light, which aims to avoid some high-order modes radiating out of the fiber and causing loss [25,26].

It is also crucial that the transmittance of the fibers should maintain a good uniformity, which is the premise of undistorted measurement and needs to be tested experimentally. The light source used in the test is expected to have a uniform cross-section covering all fibers’ input surface, which is conducive to the observation of transmittance differences; secondly and most importantly, the image captured by the camera can be used to accurately characterize fiber transmittance.

**Figure 1:**
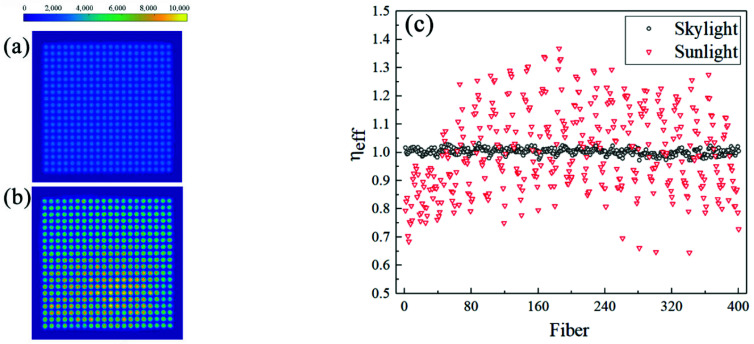
Array spots: (**a**) array spots with skylight; (**b**) array spots with direct sunlight; (**c**) the contrast of the *η_eff_* of the two types of spots: the fluctuation of *η_eff_* with direct sunlight, as the light source is much larger than that with skylight. The number of the fiber is defined as follows. The number increases one by one in each row in the array from left to right and increases in each column from top to bottom.

Two light sources are used to test transmittance—uniform diffuse light (skylight) and uniform parallel light (direct sunlight, which has a negligible actual divergence angle in a small receiving area)—and to observe the image of the output surface of the array taken by the camera. The results are quite different, as shown in Figure 1a,b. We perform average pooling processing on the spot shot by the camera with grids positioned by the output faces of fibers and use the parameter *η_eff_* to characterize the uniformity of fiber transmittance, as shown in:

(1) $$\;\eta_{eff}=\frac{ADU_{i}}{ADU_{ave}},i=1,2,\ldots ,400,$$

where *ADU_i_* is the total value of the analog digital unit (ADU) in the ith grid. The array composes 20 × 20 fibers, and *ADU_ave_* is the average value of 400 units. As shown in Figure 1c, the relative difference in the *ADU_ave_* per grid is less than 6% for uniform diffuse light, while it reaches 40% for uniform parallel light. The comparison shows that the transmittance of the array fibers should be characterized by the matrix of *ADU_ave_* from uniform diffused light, which has a good consistency; otherwise, Figure 1a will not be so ideal. Furthermore, the less-than-ideal result is believed to be caused by the introduction of imaging errors, which will be explained in detail in the next paragraph. The reason for using the difference in the matrix of *ADU_ave_* of uniformly diffused light to express the difference in fibers’ transmittance is as follows: the diffused input results in the uniform output divergence angle to ensure the fidelity of the measured spot distribution. Specifically, the input diffused light contains multiple modes, with some modes being mixed fully when transmitting in the core if the fiber is bent enough and its length is much greater than the core diameter [27,28]. Other modes, which do not meet the total reflection condition, are coupled to the cladding and disappear. Consider that multiple modes represent multiple angles, and the maximum angle relative to the sidewall section of the core is approximately the critical angle of total reflection *θ_m_*′. Therefore, the output of each fiber should be regarded as the divergent light filled with *θ_m_*, while the relationship between *θ_m_*′, *θ_m_* and NA is shown in the following:

(2) $$NA=n_{0}\sin\theta_{m}=n_{1}\sin\theta_{m}’=\sqrt{n_{1}{}^{2}-n_{2}{}^{2}},$$

where *n*_0_, *n*_1_, and *n*_2_ are the refractive indices of the incident medium, core, and cladding, respectively.

However, our system ultimately needs to measure not diffuse light but a laser beam with a slight divergence angle close to parallel. Therefore, the unsatisfactory results of uniform parallel light need to be studied. Different from the uniform diffuse light, the mode mixing in the fiber core is not complete after the uniform parallel light incident, and the degree of mixing depends on each fiber’s bending degree. When flexible fibers of the same length are arranged in a cone, they are bound to bend with an irregular and uncontrollable curvature. Then, the difference in bending will lead to the difference in the output of the divergence angle of the fiber. Regarding this result, let us combine the phenomenon reported by Gambling [29] to illustrate. For the He-Ne laser incident at 6°, the far-field output distribution of the straight fiber (*θ_m_* = 27.4°) is a ring. Suppose applying lateral force causes the bending of the fiber. In that case, the ring width gradually increases as the curvature increases and becomes a solid circle with an outer diameter more significant than the initial ring. The far-field distribution characterizes the divergence angle of the output beam. The result means that the curvature will affect the divergence angle complexly. For an ideal camera imaging system with a lens, as long as the output surface of the array is imaged through the camera, the difference in the output divergence angle does not seem to affect the image captured. This effect is enormous, to the extent that the measured results deviate by 40% from the actual output, as shown in Figure 1b,c. The reasons are as follows: 1. The difference in divergence angle leads to the difference in the amount of incoming light of the camera, limited to the field diaphragm. 2. The focal planes of the output of each fiber cannot be unified, and the diffused spot generated by defocusing is on the image plane. In addition, the degree of diffusing is different and is not easy to determine. When calculating the intensity distribution of the captured images in the pooling grids, some errors are difficult to eliminate simply by using the algorithm. Brighter spots appear more frequently in the lower half of the array, as shown in Figure 1b. Considering that the small output end is located in the lower half of the receiving surface in the actual system, there is reason to believe that the relatively large radius of curvature brings a smaller output divergence angle to this area, resulting in a higher intensity of the shot spots.

This section first verifies that the difference in the transmittance of the array fibers is less than 6%. This error can be included in the calculation of measurement uncertainty as a micro value or be corrected by the spatial non-uniformity of the units. In addition, the imaging error caused by the inconsistency of the output divergence angle of each fiber is also introduced when measuring the laser beam. This source of error has excellent influence, and we discuss its solution in Section 2.2.

### 2.2. Fiber-Homogenizer Model

**Figure 2:**
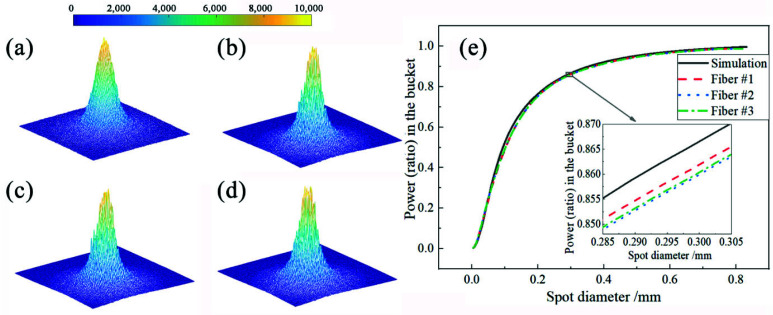
Simulated and experimental results: (**a**) simulated spot; (**b**) experimental spot of fiber #1; (**c**) experimental spot of fiber #2; (**d**) experimental spot of fiber #3; (**e**) PIB curves of four spots.

For the purpose of resolving the significant source of error mentioned in Section 2.1, a closely connected homogenizer is added at the output surface of the fiber array and shoot the spot imaging on the output surface of the homogenizer. Theoretically, for beams with different divergence angles from fibers, an ideal homogenizer can convert them into diffuse light with uniform divergence angles and present a highly similar degree of diffusion of spots on its surface. At this time, a relative model is available to include the diffused spots that belong to different divergence angles. Therefore, the crosstalk between the spots becomes regular and predictable, providing the possibility of computational correction. After the correction, the intensity distribution of the array spots can be regarded as the actual output of the fiber array without distortion. To prove the feasibility of the fiber-homogenizer model, it is first necessary to verify whether the selected homogenizer is a good Lambertian material. Although the usual light resource is a laser beam with a variable incident angle, the single-fiber output light is more suitable to achieve values close to the existing system. After all, the laser beam cannot replace the fiber output for which the incident angle is uncertain. We can discuss the homogenizer’s optical performance by comparing the experiment and simulation results. The optical model of the homogenizer can be established [30], and the Monte Carlo tracing simulation can be performed with the parameters of the fiber and Lambert’s law in the following form:

(3) $$I_{\theta }=I_{N}\cos\theta ,$$

where *I_N_* is the output light intensity in the normal direction of the homogenizer surface, and *I_θ_* is the output light intensity in the direction of the angle *θ* with the normal. A 1064 nm laser is taken for normal incidence and three fibers with large bending differences are randomly selected for experiments. Using three-dimensional graphs and PIB curves to characterize simulated and experimental spots, as shown in Figure 2, the RMSE of PIB curves belonging to the simulated spot (a) and the three experimental spots (b), (c), and (d) are very low, being 1.10%, 1.16%, and 1.15%, respectively, proving that our homogenizer can be seen as a Lambertian material. In addition, the PIB curves of spots (b), (c), and (d) are almost overlapped, which can be seen in Figure 2e. A uniform Gaussian model is, therefore, feasibly able to simulate 400 homogenized spots.

## 3. Methods

### 3.1. Instrument Design

The instrument design includes a high-reflectivity target plate, fiber array, filter, attenuator, homogenizer, CCD camera, packaging structures, and data-processing system. The array composes 400 flexible equal-diameter multimode step-type fibers in 20 rows and 20 columns, and each one is perpendicular to the array face within a certain distance. The theoretical NA of the fiber is 0.22 by Equation (1); the core diameter is 0.105 mm, and the cladding diameter is 0.125 mm. The spacing of fibers at the beam-receiving face is 6 mm, while the sampling resolution still has much room to improve, considering that the outer diameter of each fiber does not exceed 1 mm. Bare fibers arrange the output with the coating stripped. It is necessary to maintain a small spacing to achieve a sufficient ratio to shrink the beam. At the same time, it is necessary to prevent the crosstalk from being too large to eliminate after the spacing is determined to be too small. The final design value is 0.150 mm. The arrangement of the two faces is rectangular, and the positioning is one-to-one. The outer diameter of the receiving end is 114 mm, and the beam is shrunk by 40 times in diameter. The applicable wavelength band of the fiber is 600~1800 nm, and the corresponding filter, attenuator, and CCD camera can be set according to the desired band.

**Figure 3:**
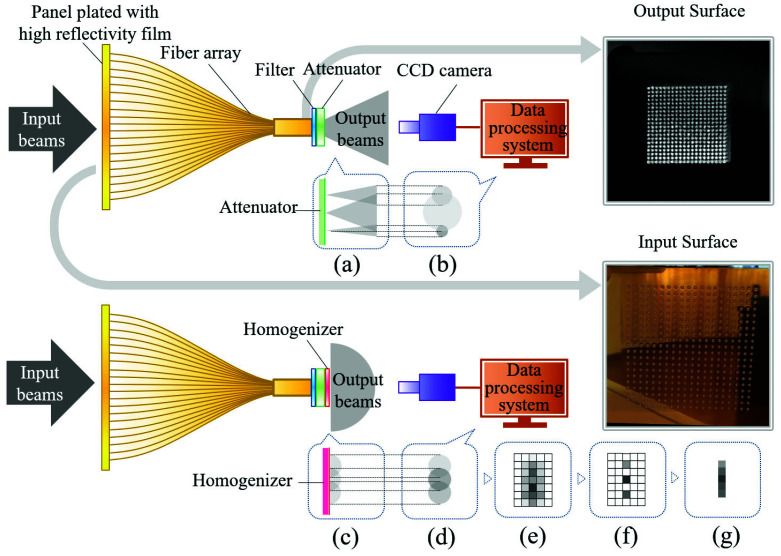
System diagram: the upper half of the diagram shows the light transmission process without the homogenizer (part of the spots of the array), while the lower half shows the process with the homogenizer. (**a**) Different divergence angles of the output array beams without the homogenizer; (**b**) captured array spots with mutual crosstalk that are not easy to divide; (**c**) output beams that become array diffusers; (**d**) captured array spots with mutual crosstalk that can be predicted by the model and removed by the algorithm; (**e**) array spots after positioning and gridding; (**f**) array spots after crosstalk removal; and (**g**) restored spots.

The working principle of the system is shown in Figure 3. First, laser beam incidents occur on the sampling target plate, most of the light is reflected out of the system to reduce the total amount of converted heat, and the fiber ferrules are connected to the array holes on the plate. Then, the cross-section spot is sampled as an array of many small spots and transmitted to the output end of the fiber array; at this time, the total diameter of the origin spot decreases as the array becomes more compact. After filtering and attenuation, in order to prevent the shooting distortion shown in Section 2.1, the array spots are homogenized. The homogenizer is made of a kind of bulk-scattering material to achieve sufficient homogenization, but to avoid the spot crosstalk being too much, its thickness is set at 0.1 mm. As shown in Figure 3d, the output beams with different divergence angles are converted into diffuse beams after homogenization. Meanwhile, the array spots are diffused and overlapped with the surrounding spots. For each unit spot, although the total intensity is unknown, they all follow the known uniform Gaussian model, as performed in Section 2.2. Therefore, the array spots with crosstalk can be divided by computational processing to restore the real output of the fiber array. Then, coupled with interpolation expansion processing, the original cross-section spot can be restored, and the power density distribution is finally made available through this and the power calibration coefficient.

### 3.2. Computational Correction

**Figure 4:**
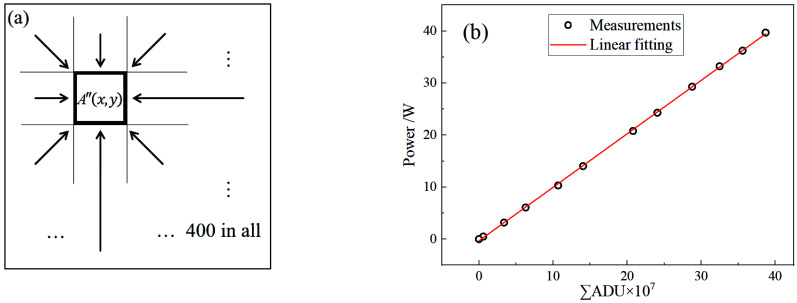
(**a**) Schematic diagram of mutual crosstalk between spots; (**b**) calibration curve of total power.

The CCD camera shoots 400 spots with mutual crosstalk. Each spot is affected by others, and up to 400 units can be considered, including itself, as shown in Figure 4a. This crosstalk can be analogized to convolution, and Section 2.2 determines a uniform unit spot model, which can also be analogized to the convolution kernel, and the influence of each spot on others can be quantified as a linear function of its total intensity. An idea similar to deconvolution is proposed in order to solve this problem. Therefore, a set of four-hundred-element linear equations can be set up. The known quantity is the measured intensity of each grid determined by the corresponding fiber, and the solution quantity is the actual output of each fiber without crosstalk. The number of unknowns and equations are both 400, so the equations are solvable. The following is the specific solution.

Initially, positioning each fiber in the image is needed before the actual measurement and after relative fixing between the fiber array and the camera. It is suitable and convenient to take natural light as the light source, and to shoot the array face directly, without filter and homogenizer in order to obtain the positioning image *L_m_*_×*m*_, where *m* × *m* is the camera resolution. Secondly, the experimental spot provided in Section 2.2 is used as the model. Assuming that the unit in the first row (column) can affect that in the 20th row (column) in the farthest case, the spot model needs to be processed with average pooling by grids of 39 × 39 to obtain *P*_39__×39_. Thirdly, *L_m×m_* is used to filter the measured image *A_m×m_* and averagely pool it to obtain the matrix *A*′_20__×20_ as a known quantity and solve the actual spot *A*′′_20__×20_ by:

(4) $$A’’(x,y)=\sum_{i=1}^{20}\sum_{j=1}^{20}\left((A’(i,j))P(20+x-i,20+y-j)\right),$$

The solution of Equation (4) is:

(5) P’(i,j)=P(20+x−j,20+y−j),i=1,…,20;j=1,…,20,

(6) $$P{’}_{20\times 20}\overset{reshape}{\to }P{’}_{1\times 400},$$

(7) P’’(1:400,(x−1)×20+y)=P’,i=1,…,20;j=1,…,20,

(8) $$A’{’}_{1\times 400}P’{’}_{400\times 400}=A{’}_{1\times 400},$$

where *P*′ and *P*′′ are the parameters. After the crosstalk is removed, *A*′_1__×400_ is reshaped into *A*′′_20__×20_, and the actual output of 400 fibers can be obtained.

Then, the interpolation method to expand the beam is used. Suppose the resolution of the restored spot is required to be *l* × *l*. In that case, the number of interpolated pixels for every two-unit interval is *n* = (*l* − 20)/21, and per-pixel size *s* = (*D* × 19)/*l*, where D is the sampling interval of the beam-receiving face of the fiber array, and the distance between the centers of two adjacent fibers. Suppose it is desired to visually show the effect of sampling on the spot intensity distribution. In that case, the pixel size can equal to the light entrance aperture *Φ* of each unit in the receiving face. At this time, *n* = 2*D*/(*π*^0.5 × *Φ*). Specifically, the bicubic nearest-neighbor interpolation is chosen, which can be calculated by:

(9) $$\upsilon (x,y)=\sum_{i=0}^{3}\sum_{j=0}^{3}a(x,y)x^{i}y^{j},$$

where *υ*(*x*, *y*) is the gray value of a single pixel and *a*(*x*, *y*) is the coefficient calculated by the 16 nearest pixels.

After the relative intensity distribution is obtained, the power density distribution needs to combine the total power in order to be solved. A 1064 nm laser source is used to test the system; it is found that the total ADU of the measured image is linearly related to the total power measured by the power meter in Figure 4b, and the coefficient of the determination of linear fit is 0.99979. In this case, the slope *γ* is 1.0282 × 10^−7^, and the total power *P_sum_* can be solved as

(10) $$P_{sum}=\gamma \times ADU_{sum},$$

where *ADU_sum_* is the total value of the image’s ADU. Furthermore, power density *I* can also be calculated by *γ*.

(11) $$I=\frac{\gamma \times ADU}{s^{2}},$$

## 4. Results and Discussions

This article reports a beam quality measurement technology based on cone-arranged fibers and the corresponding computational correction. The related system achieves a resolution of 6 mm, a measurement aperture of up to 114 mm, and a suitable wavelength range of 600~1800 nm. After performing a 40-fold reduction in the diameter of the beam by fiber array, the CCD camera only needs to shoot the object less than 3 mm in length, while the distortion can be ignored with no need for a large field of view. In order to verify the accuracy of the technology, a large-size Gaussian spot was shrunk, photographed, and restored, then compared with a photograph of the actual spot taken by the diffuse reflection screen in the same axial position. In this case, the parameters of the laser and other instruments are shown in Table 1.

**Figure 5:**
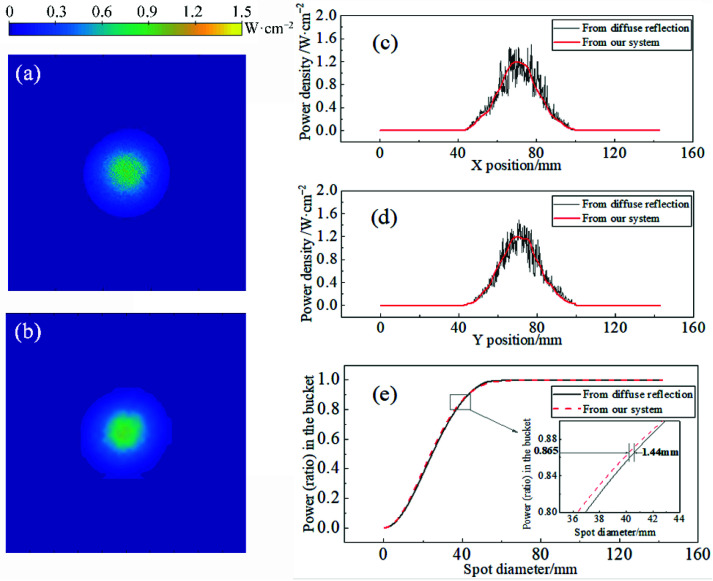
(**a**) Actual spot collected by the diffuse reflection screen; (**b**) spot measured by the system; (**c**) centroid section lines of the two types of spots in the x-direction; (**d**) centroid section lines of the two types of spots in the y-direction; (**e**) PIB curves of the two types of spots.

It should be noted that the spot imaged by the diffuse reflection screen is superimposed with small noise and appears grainy, as shown in Figure 5a,c,d, which is not a feature of the actual spot, but is caused by the scattering properties of the screen, which is a limitation of this method. The light source is stable because the jitter range of the centroid in the x- and y-directions does not exceed 0.1 mm in the test. Therefore, the two types of spots are averaged in 100 frames, as shown in Figure 5. Obviously, the measured spot (b) is very similar to the actual spot (a), and their PIB curves have a high degree of coincidence, as shown in Figure 5e, while the RMSE is 0.85%. Additionally, the diameters of the 86.5% PIB of the two spots are only 0.44 mm apart, which is less than 6 mm of the sampling resolution, meaning that only the error caused by the sampling resolution needs to be analyzed when measuring the beam width. Another standard indicator of the laser is the centroid, which is located at 71.00 mm in the x-direction and 71.42 mm in the y-direction in the measured spot, and at 70.71 mm and 71.00 mm in the actual spot. The difference between the centroid positions of the two spots is no more than 1 mm, about 2.5% of the spot diameter. This error may result from resolution differences between the diffusely reflected system and our system. Additionally, in Figure 5c,d, it can observe that the centroid section lines of each direction all correspond well, and the measured spot is smoother, which makes up for the limitation of the grainy spot caused by the diffuse reflection screen. Moreover, its high-reflectivity-receiving target surface makes the most of the laser out of the system by specular reflection. Compared with diffusely reflected light, the specularly reflected light will not scatter everywhere, which is easier and safer to handle.

**Figure 6:**
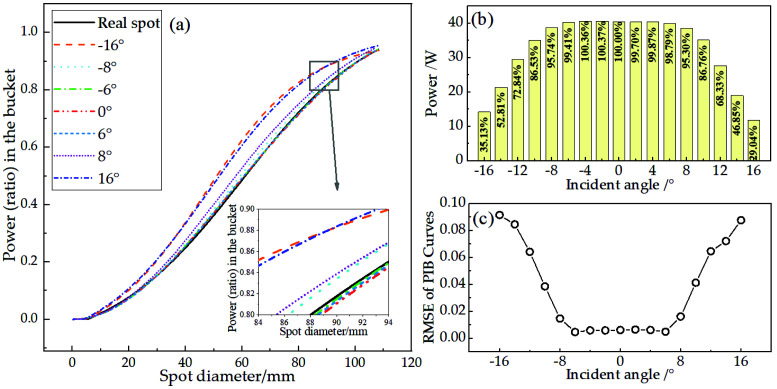
(**a**) PIB curves of the measured spots at different incident angles and the actual spot; (**b**) absolute value of the measured total power and their ratios relative to the true power when the incident angle changes between −16° and 16°; (**c**) RMSEs of the PIB curves of the measured spots and the actual spot.

Since the incident angle to the target may change due to the jitter of the loading platform during actual measurement, it is of significance for the system to stabilize the measurement result within a certain incident angle range. The stable beam with an incident angle between −16° and 16° was measured, which is shown in Table 1, with a constant distance to the target; the results are shown in Figure 6b. The change in the measured total power is less than 2% at −6°~6° and less than 5% at −8°~8°. Additionally, in Figure 6a,c, a similar rule also shows up in the power density distribution: the RMSEs of the PIB curves of the measured spot and the actual spot are all lower than 1% at −6°~6° and lower than 2% at −8°~8°. This error is caused by the small difference in the coupling efficiency of the fiber array when the angle changes generally. It can be considered in the measurement uncertainty of the spot centroid and diameter. Its weight is equal to the response error of CCD, referred to by [31], and given by calibration. According to the corresponding relationship between the error and the incident angle, the applicable incident angle range of the system can be derived by the measurement requirements. Because of the accurate measurement of the beam width, we have reason to believe that the measurement of the profile of other non−Gaussian beams, such as Bessel beams, is also possible, as long as the spot covers enough sampling fibers.

Considering that the theoretical NA of the fiber is 0.22, the *θ_m_* is calculated as 12.7°, according to Equation (2). However, the power is reduced to 68.3% and 72.8% of the actual power (normal incident) at 12° and −12°, respectively. It is known that the application object of theoretical NA and *θ_m_* is the meridian ray in a straight fiber, which is the ray in any plane through the central axis of the fiber. In actual application scenarios, the full incidence at *θ_m_* and the bending of the fiber will cause the light to be refracted into the cladding and become gradually lost there. In addition, the RMSE increased to approximately 6% at this angle, indicating that the loss is non−uniform for fibers, which is caused by the difference in the bending shape of each fiber. In summary, *θ_m_* can only be used as a reference for the system’s allowable incident angle. However, when switching to the fiber with a larger theoretical NA, the allowable incident angle of the system will be positively affected as the critical incident angle that meets the total reflection condition is increased. In addition, the system has good performance in the range of measurable laser beam width and light intensity; for the former, the maximum is 66.7% of the array aperture (Gaussian beam), which is generally 76 mm. In this case, the power received by the array reaches 99% of the total power of the laser beam [31]. As for the minimum, the spot needs to cover at least ten fibers in the radial direction so as not to affect the subsequent interpolation calculation and spot recovery [32]; therefore, the minimum laser beam width needs to reach 33.4% of the array aperture, that is, 38 mm. Regarding the allowable light intensity, the minimum is in the order of mW/cm^2^, taking into account the data in the power density calibration in Section 3.2; the maximum is in the order of kW/cm^2^, taking into account the test experiment of the total reflection metal target used and the laser damage threshold of the sampling fibers [33].

## 5. Conclusions

This paper proposes a new technology for laser parameter measurement based on an array of cone−arranged fibers. The system and the computational correction have been established and verified through theoretical and experimental analysis. The results show that the measured spot corresponds well to the actual spot in terms of power density distribution, centroid position, and centroid jitter, providing a basis for accurately calculating multiple laser parameters. The advantages of this technology are summarized as follows:

1. The CCD camera can shoot large spots without the distortion caused by a large field of view for the cross−section spot is shrunk with high fidelity.
2. The sampling resolution is higher and the design is simpler compared with the traditional array target.
3. The allowed incident angle range is acceptable. The measurement of the total power and the power density distribution of the spots has high accuracy when the beam’s incident angle is between −8° and 8°.

Here are some ideas regarding future work:

1. Measurement resolution can be improved further without the limitations of complex unit structure. For example, the hexagonal fiber layout will be an optimization direction for the higher fiber packing density than the square layout.
2. When it is needed to focus on a larger angle, a fiber with higher NA should be considered. The system’s actual NA and the fiber’s theoretical NA are predicted to have a linear correlation, and the relevant theoretical discussion and experimental verification are worth studying.

## Figures and Tables

**Table 1:** Experiment instrument parameters.

Instrument	Feature	Specification
Laser	Wavelength	1064 nm
	Polarization	Linear
	Output power	1~100 W, continuously adjustable
	Working mode	Continuous wave
Diffuse reflection screen	Diffuse reflectance ratio	>99%
	Material	Thermoplastic resin
Filter	Central wavelength	1064 nm
	Bandwidth	8 nm
Attenuators	Type	Absorptive neutral density
	Optical density (OD)	2~5
Lenses	Focal for actual spot	12 mm/F 1.4
	Focal for reduced spot (with extension ring)	50 mm/F 1.4
CCD camera	Camera	Allied Vision Prosilica GT
	Sensor	Sony ICX674ALG
	Pixels	1936 (H) × 1456 (V)
	Pixel size with lens for actual spot	121 μm (H) × 121 μm (V)
	Pixel size with lens for reduced spot	5 μm (H) × 5 μm (V)
